# Supplementary material for: Splice-Junction-Based Mapping of Alternative Isoforms in the Human Proteome
Source: Cell Rep. Author manuscript; Available in PMC 2020 Jan 15. (PMC6961840; doi:10.1016/j.celrep.2019.11.026)
Supplement: 3 [file NIHMS1546469-supplement-3.zip › DF2/PXD009021/Liver-42-P31994-ISANPTNPDEADK.pdf]

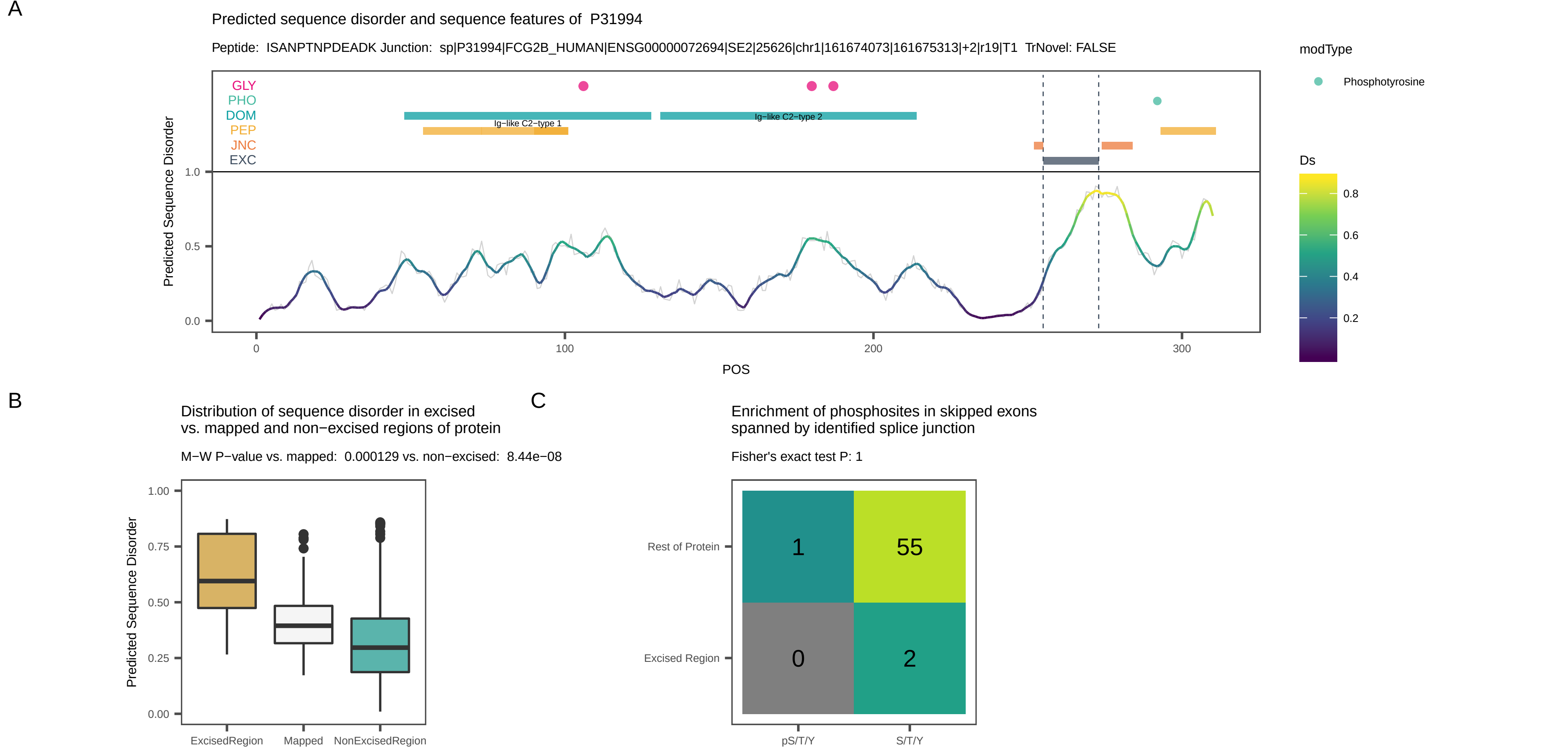
A
Predicted sequence disorder and sequence features of P31994
Peptide: ISANPTNPDEADK Junction: sp|P31994|FCG2B_HUMAN|ENSG00000072694|SE2|25626|chr1|161674073|161675313|+2|r19|T1 TrNovel: FALSE
GLY
PHO
DOM
PEP
JNC
EXC
Ig−like C2−type 1
Ig−like C2−type 2
Predicted Sequence Disorder
1.0
0.5
0.0
0
100
200
300
POS
modType
Phosphotyrosine
Ds
0.8
0.6
0.4
0.2
B
Distribution of sequence disorder in excised
vs. mapped and non−excised regions of protein
M−W P−value vs. mapped: 0.000129 vs. non−excised: 8.44e−08
Predicted Sequence Disorder
1.00
0.75
0.50
0.25
0.00
ExcisedRegion
Mapped
NonExcisedRegion
C
Enrichment of phosphosites in skipped exons
spanned by identified splice junction
Fisher's exact test P: 1
Rest of Protein
Excised Region
1
55
0
2
pS/T/Y
S/T/Y
